# Conducting Polymers as Versatile Tools for the Electrochemical Detection of Cancer Biomarkers

**DOI:** 10.3390/bios13010031

**Published:** 2022-12-27

**Authors:** Jincymol Kappen, Małgorzata Skorupa, Katarzyna Krukiewicz

**Affiliations:** 1 Centre for Nanoscience and Nanotechnology, Department of Chemistry, The Gandhigram Rural Institute-Deemed to be University, Dindigul 624 302, Tamilnadu, India; 2 Department of Physical Chemistry and Technology of Polymers, Silesian University of Technology, M. Strzody 9, 44-100 Gliwice, Poland; 3 Joint Doctoral School, Silesian University of Technology, Akademicka 2A, 44-100 Gliwice, Poland; 4 Centre for Organic and Nanohybrid Electronics, Silesian University of Technology, S. Konarskiego 22B, 44-100 Gliwice, Poland

**Keywords:** biomarkers, biosensors, cancer, conducting polymers, electrochemical sensors

## Abstract

The detection of cancer biomarkers has recently become an established method for the early diagnosis of cancer. The sensitive analysis of specific biomarkers can also be clinically applied for the determination of response to treatment and monitoring of disease progression. Because of the ultra-low concentration of cancer biomarkers in body fluids, diagnostic tools need to be highly sensitive and specific. Conducting polymers (CPs) are particularly known to exhibit numerous features that enable them to serve as excellent materials for the immobilization of biomolecules and the facilitation of electron transfer. Their large surface area, porosity, and the presence of functional groups provide CPs with binding sites suitable for capturing biomarkers, in addition to their sensitive and easy detection. The aim of this review is to present a comprehensive summary of the available electrochemical biosensors based on CPs and their composites for the ultrasensitive detection of selected cancer biomarkers. We have categorized the study based on different types of targeted biomarkers such as DNAs, miRNAs, proteins, enzymes, neurotransmitters and whole cancer cells. The sensitivity of their detection is enhanced by the presence of CPs, providing a limit of detection as low as 0.5 fM (for miRNA) and 10 cells (for the detection of cancer cells). The methods of multiplex biomarker detection and cell capture are indicated as the most promising category, since they furnish more accurate and reliable results. Ultimately, we discuss the available CP-based electrochemical sensors and promising approaches for facilitating cancer diagnosis and treatment.

## 1. Introduction

Cancer is undoubtedly one of the leading reasons for global deaths. In 2020,, there were 4 million new cases of cancer and 1.9 million cancer-related deaths reported in the European Union [1]. Since cancer is a result of an uncontrolled proliferation of misregulated cells, it is a challenge to detect it at the early stage of the disease, particularly in the asymptomatic stage [2]. The progression of cancer disease in the body does not only alter a specific organ or tissue, but has severe effects on the whole organism by modifying the chemical composition of different body fluids. Consequently, these changes may be used to distinguish between an affected person and a healthy individual.

**Scheme 1:**
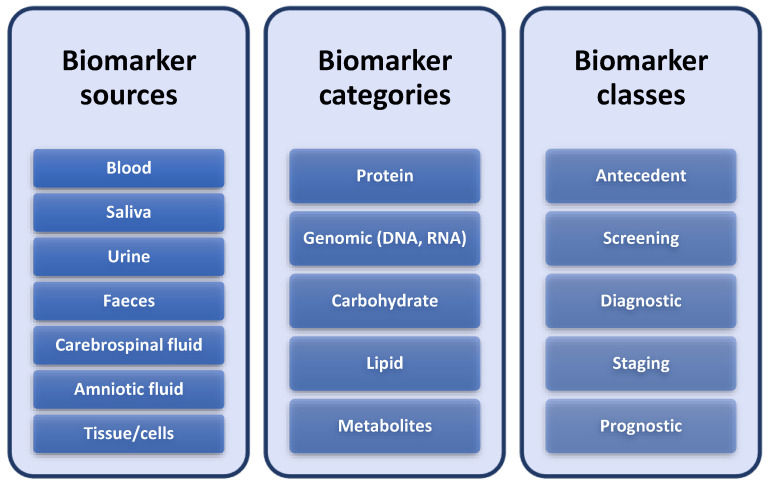
Categorization of biomarkers’ sources, categories, and classes.

Cancer biomarkers are biological molecules of different origin that can be found in blood, tissue or other body fluids (Scheme 1). Biomarkers can be categorized based on their biological identity as proteins, genomic biomarkers (DNA and RNA), carbohydrates (glucose, sucrose, glycans), lipids (cholesterol, prostaglandins, phospholipids), and metabolites (reactive oxygen species, dicarboxylic acid, inorganic ions). Biomarkers can be strictly associated with the metabolic activity of cancer cells or highlight the genetic tendency of the organism to develop cancer [3]. Therefore, the analysis of biomarkers can be used to either predict a risk of cancer (anteceding and screening biomarkers), help in cancer diagnosis (diagnostic and staging biomarkers), or inform about a likely cancer outcome (prognostic biomarkers).

Despite current advancements in genomics, proteomics and bioinformatics, leading to the identification of a plethora of potential biomarkers, there are many barriers affecting the transfer of results from a lab bench to medical practice [4]. The major challenges in the field of bioanalytics are associated with deficiencies in sensitivity, specificity, and predictive values of analytical methods. Because of the ultra-low concentration of cancer biomarkers, diagnostic tools need to be highly sensitive and specific. Enhanced sensitivity of a biosensor is, therefore, crucial to find out the ultra-small concentration of the biomarker in the early diagnosis and variation in the concentration of the biomarker during the prognosis to get a picture on the progression of treatment. Consequently, researchers have devoted much attention to making specific biosensors for each target with maximum sensitivity in a practical assay. Due to the heterogeneity of cancer, the concentration of a particular biomarker may vary between different patients and different stages of the disease. Therefore, a recent trend suggests the design of analytical platforms allowing for the simultaneous detection of multiple biomarkers and the analysis of their mutual relationships.

The detection of cancer biomarkers can be clinically applied not only for the early detection of cancer and accurate pre-treatment staging, but also for the determination of response to treatment and monitoring of disease progression [5]. Even though there are numerous therapies that can be applied to cancer patients (surgical resection, chemotherapy, radiotherapy, targeted therapy, immunotherapy, hormone therapy, etc.), it is difficult to predict which one will be the most effective for a particular patient. Accordingly, a sensitive biomarker detection system, available at the point-of-care, would be indispensable in the assessment of the efficacy of treatment. For this purpose, conducting polymers (CPs) have recently emerged as promising and versatile materials with remarkable properties suitable for biosensing applications. Exhibiting biocompatibility, developed surface area and mechanical flexibility, CPs provide improved signal transduction, which allows for the increase in sensitivity of analysis [6]. Additionally, a wide range of immobilization protocols of various recognition elements makes CPs versatile carriers applicable for the design of multiplex arrays for biosensing applications [6,7,8,9].

The aim of this paper is to present the recent advances in the development of electrochemical biosensors, particularly those based on CPs, allowing for the ultrasensitive detection of cancer biomarkers.

## 2. Electrochemical Biosensors

**Scheme 2:**
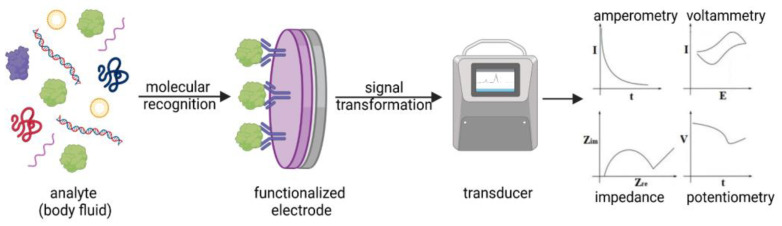
Schematic diagram showing the working principle of an electrochemical biosensor. Created with BioRender.com.

Electrochemical methods, including potentiometry, coulometry, voltammetry, amperometry and impedance spectroscopy, are standard laboratory techniques used for the qualitative and quantitative analysis of substances. Electrochemical analysis is based on the measurement of potential, charge, current or conductivity values to determine the concentration of the analyte or its electrochemical reactivity. Electrochemical biosensors act in a similar way, i.e., through the use of functionalized electrodes able to produce an electrical signal when a particular biological entity is attached (Scheme 2). Both the specificity and selectivity of the electrochemical biosensor are determined by a molecular recognition element (enzyme, antibody, nucleic acid, etc.) that needs to be appropriately immobilized on the surface of the electrode to avoid its deactivation [10,11]. On the other hand, the sensitivity of the biosensor is greatly influenced by the transducer (electrode) and its modifications.

Among the various electrochemical measurement methods, four are the most commonly used in the design of biosensors [11,12,13]. Amperometric biosensors are the best-known devices whose mode of action is based on the oxidation or reduction of an electroactive biological element that produces a detectable current signal. Potentiometric biosensors, on the other hand, allow an electrical potential difference between two electrodes to be quantified when the cell current is zero. This allows of the use of the Nernst equation to relate the concentration of an analyte to a potential difference. Biosensors take the advantage of different voltammetric techniques (differential pulse voltammetry: DPV, cyclic voltammetry: CV, square wave voltammetry: SWV, linear sweep voltammetry: LSV), use a varying potential to measure resulting current, and provide analytical information with low cost, good selectivity, and high sensitivity. Impedimetric biosensors use the variation in electrode impedance to detect the presence of analytes with low excitation voltage, fast speed, and high sensitivity, making them particularly suitable for a long-term and real-time detection of biomarkers.

## 3. Conducting Polymers

**Scheme 3:**
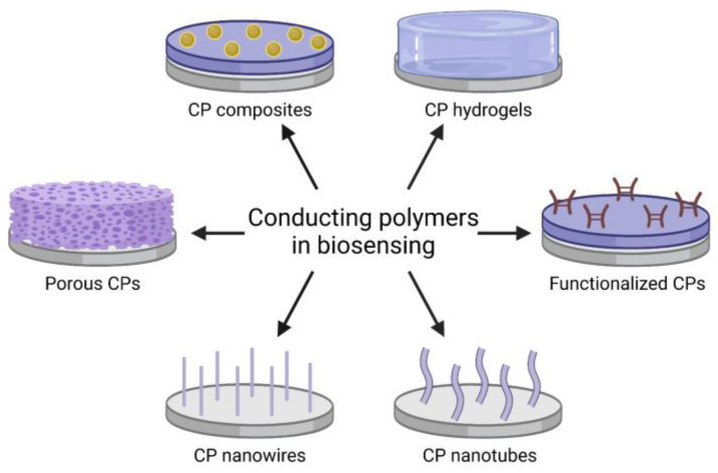
Different forms of conducting polymers (CPs) used for the design of electrochemical biosensors. Created with BioRender.com.

Conducting polymers (CPs) belong to a special class of polymeric materials that combine mechanical properties of polymers with the ability to conduct currents typical for metals. In turn, CPs have found applications in various fields, from optoelectronics and energy storage devices [14] to biomedical devices and biosensors (Scheme 3) [15]. In the latter, CPs are especially pursued due to their biocompatibility, sensitivity, improved signal transduction, porosity, and mechanical flexibility [6].

Electrical conductivity in CPs results from the presence of a conjugated backbone, consisting of alternating single and double bonds throughout the whole structure. σ-bonds present in the single and double bonds are highly localized, whereas π-bonds are weaker and more delocalized. The conjugated system is formed by the overlap of p-orbital electron clouds in the series of π-bonds, which promotes an easier delocalization of electrons and therefore facilitates their mobility, providing a pathway for charge carriers [16,17,18]. However, in the neutral state, CPs are only weakly conductive [6]. Another important factor required for an efficient charge conduction through polymer chains is the introduction of doping ions into their structure and creating self-localized excitations (i.e., solitons, polarons or bipolarons) in the conjugated chains [18]. The process of CP doping corresponds to redox reactions within the polymer matrix and the stabilization of the backbone. In the case of oxidation (p-doping), the doping ions take the form of anions or electron donors, whereas reduction (n-doping) involves the use of electron acceptors–cations. The p-doping is generally more common due to the fact that it provides more stable charge carriers than the negatively charged forms of CPs [19].

The electronic structure of CPs is sensitive to changes in the polymer chain conformation and in the environment, e.g., during biological recognition. These changes manifest themselves in altered conductivity and spectroscopic properties that can be used directly for signal detection [20]. Doping/dedoping processes can be also used to reversibly change electronic and optoelectronic properties of CPs [21]. Another advantage of CPs is their dual mechanism of conductivity. Since CPs have the ability to transport both electronic and ionic charges, they can be used to efficiently convert an ionic signal into a solid state electronic signal [22].

CPs provide a variety of immobilization pathways for recognition elements. The simplest mechanism is a physical adsorption, in which biomolecules become immobilized on the polymer surface by electrostatic interactions, hydrogen bonds, van der Waals forces, or hydrophobic interactions [6,9]. The chemical functionalization of CPs’ backbone can be used to provide stronger physical interactions for even better immobilization and contact with the CP. Polymer chains can be modified at the monomer level [23] or during bulk processing after polymerization, which is mostly applied to chemically synthesized CPs [22]. Additional functional groups can either improve the affinity of the bioreceptor to CPs, or be used to further increase the binding efficiency by a covalent attachment. Covalent immobilization is a well-known and effective technique used to improve the stability, activity, and sensitivity of biosensors, and to prevent the leaching of the molecular recognition element [24].

The unique immobilization mechanism associated with CPs is the ability to confine the biosensor elements within the volume of the CP matrix. This can be achieved by simply adding biomolecules to the solution intended for electrochemical polymerization. In this way, selected molecules, such as peptides, enzymes and antibodies, can act as dopants and become entrapped in the polymer matrix [8]. It is a one-step process that offers good reproducibility and control of film thickness [6]. Although the efficiency of target binding can be reduced due to the partial burial of the bioreceptors and diffusion constraints [9], CPs are characterized by a developed specific surface area. By tuning polymerization conditions [18] or using organic molecules as chain ‘connectors’ [25], porosity can be controlled so that biochemical interactions between analytes and receptors are still possible when embedded in the 3D structure of the CP [26]. This property can also increase the selectivity of the biosensor by providing a size exclusion membrane for analytes penetrating the CP network [6,26].

Some CPs are compatible with a technique of molecular imprinting. In this method, the resulting label-free biosensing platform consists only of the polymeric matrix with imprinted recognition sites based on the template of a target molecule [25]. The molecular imprinting technique provides high selectivity and limits the use of biologically active components in the final design [27]. The electrochemical polymerization of CPs allows a precise deposition of the polymer on the microelectrode sites. Consequently, it is possible to construct multiplex microelectrode arrays for biosensing applications [7]. The precise construction of biosensor chips and the multifunctionality of detection can also be achieved with inkjet printing technologies that use soluble CPs in the ink composition [28,29]. Additionally, the mechanical properties of CPs enable the development of flexible biosensors [27,29,30], which are particularly important for the transition to wearable or in vivo devices.

## 4. Conducting Polymer-Based Electrochemical Sensors for Cancer Diagnosis

**Scheme 4:**
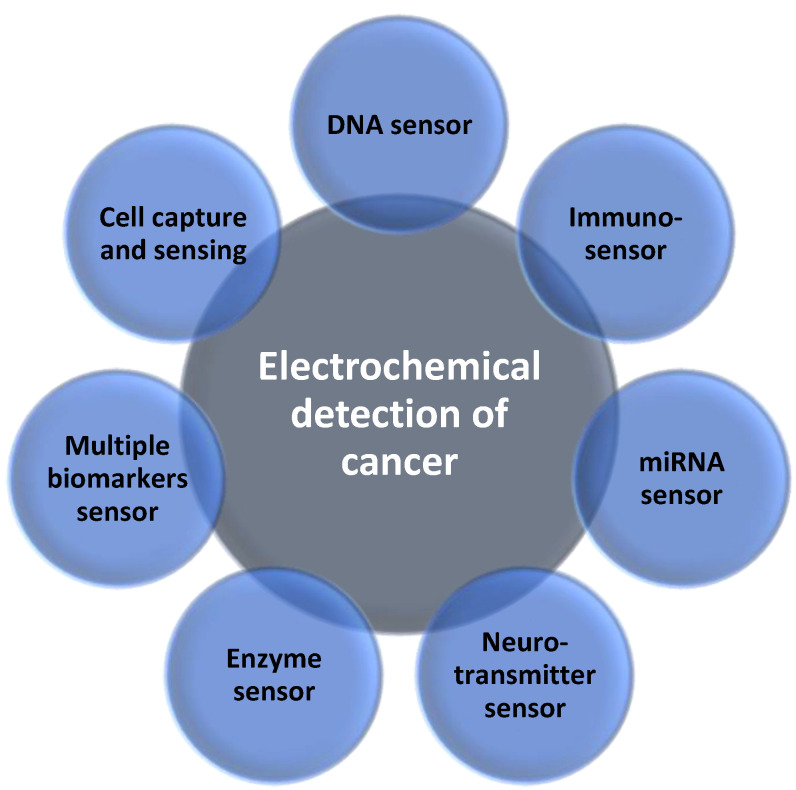
Different forms of cancer biomarkers that can be detected through electrochemical methods.

Electrochemical methods allow for the sensitive detection of cancer biomarkers present in different forms, such as DNA, proteins, miRNA, antigens, enzymes or neurotransmitters (Scheme 4) [12]. The application of CPs for the functionalization of electrodes can be used to further increase the sensitivity and selectivity of analysis.

### 4.1. DNA Sensors

**Scheme 5:**
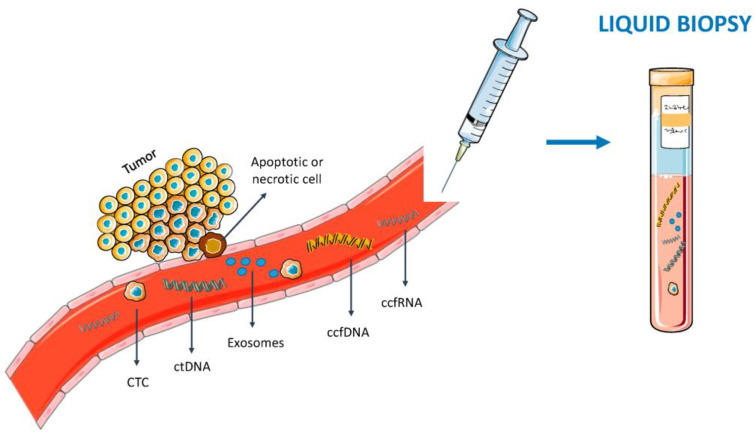
Circulating tumor cells (CTC), circulating cell-free DNA (ccfDNA) [including circulating tumor DNA (ctDNA)], circulating cell-free RNA (ccfRNA), and exosomes are released from tumor cells to the bloodstream. Hence, blood can be collected in the context of a liquid biopsy and analyzed using electrochemical biosensors. Reprinted with permission from [31]. Copyright (2020) MDPI.

Circulating tumor cells and circulating cell-free DNA (cfDNA) are important for the early detection and screening of cancer, as they can be used to identify tumors and genetic alterations (Scheme 5) [31]. The cfDNA level is comparatively higher in cancer patients than in healthy people. Therefore, it is crucial for early cancer diagnosis to detect even a small deviation from the normal cfDNA concentration. For instance, an electrochemically deposited polypyrrole (PPy)-modified chip was fabricated for the detection of cfDNA [32]. The over-oxidized surface of the chip was immersed in blood serum, which enabled the successful immobilization of DNA on the chip. The clinical utility of the chip was tested by extracting cfDNA from healthy donors and lung cancer patients.

Wang et al. [33] reported the detection of breast cancer biomarker BRCA1 using poly(3,4-ethylenedioxythiophene) (PEDOT) doped with the zwitterionic polypeptide. Polypeptide-doped PEDOT (PEDOT/PEP) was electrodeposited on the surface of a glassy carbon electrode (GCE), and GCE/PEDOT/PEP was incubated in a DNA capture solution containing the coupling agents N-hydroxysuccinimide (NHS) and 1-ethyl-3-(3-dimethyl aminopropyl)carbodiimide (EDS). Furthermore, an indicator (methylene blue, MB) was attached to the surface of the sensor electrode and immersed in target DNA for the attachment on the electrode surface. The differential pulse voltammogram (DPV) was then recorded to find out the concentration of BRCA1 indirectly from the current response of MB. Due to the strong interactions between the DNA probe and BRCA1, MB was detached from the surface, and the current response was decreased accordingly. Based on this strategy, the sensitive and selective detection of BRCA1 was carried out and, finally, the analysis of a real sample was performed with a human blood serum sample [33]. For the ultrasensitive detection of BRCA1, Shahrokhian and Salimian [34] fabricated an electrochemically reduced graphene oxide-PPy composite. The CP-coated reduced graphene oxide provided more active sites for the immobilization of DNA. The quantitative detection of BRCA1 was possible in the linear range of 10 fM–0.1 µM with a detection limit as low as 3 fM in a blood serum.

### 4.2. miRNA Sensors

**Scheme 6:**
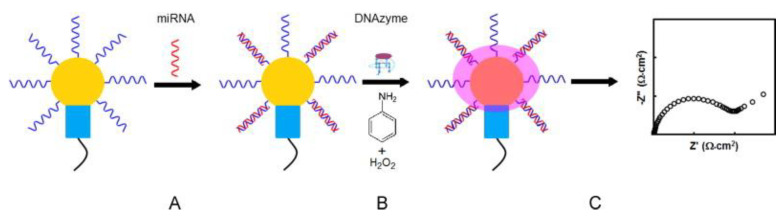
Schematic illustration of the working principle of the label-free miRNA biosensor: (**A**) miRNA hybridization, (**B**) hybridized miRNA-guided PAn deposition, and (**C**) Nyquist plot. Reprinted with permission from [36]. Copyright (2014) Elsevier.

The changes in the miRNA expression within a tissue are often associated with disease states. The involvement of miRNA in human cancer was first reported by Caline et al. [35], who described its key role in cancer metastasis. An impedimetric miRNA biosensor was fabricated based on the miRNA-guided deposition of polyaniline (PAn) from the monomer (Scheme 6) [36]. The gold electrode was then modified with a neutral peptide nucleic acid capture probe to capture miRNA, and was incubated in a solution containing aniline, hydrogen peroxide and G-qudraplex-hemin DNAzyme. The DNAzyme catalyzed the polymerization of aniline, which was guided by the miRNA standing on the electrode surface. The as-formed sensor was able to detect 0.5 fM of target miRNA.

In a similar study, the ruthenium oxide-tagged miRNAs-initiated polymerization of aniline was executed for the voltammetric quantification of miRNA in lung cancer cells [37]. A templated growth of PAn resulted in the formation of a conducting layer exhibiting a dual-dependence amplification of detected signals, greatly increasing the sensitivity of the device. The prostate cancer biomarker miR-141 was quantified using a biosensor developed from multi-walled carbon nanotubes (CNTs) and a naphthoquinone-based polymer. The sensor showed higher sensitivity with a limit of detection (LOD) of 8 fM [38]. An ultrasensitive electrodetection of miRNA was also developed based on a microelectrode through the immobilization of peptide nucleic acid (PNA) capture probes in nanogaps of a pair of interdigitated microelectrodes [39]. Hybridization was performed with their complementary target miRNA, followed by the electrochemical deposition of PAn nanowires driven by the electrostatic interaction between anionic phosphate groups in miRNA and cationic aniline molecules. The conductivity of the deposited nanowires correlated directly to the amount of hybridized miRNA. Under optimized conditions, the target miRNA can be quantified in a range from 10 fM to 20 pM with a detection limit of 5 fM. The biosensor array was applied to the direct detection of miRNA in total RNA extracted from HeLa cells and lung cancer cell lines.

### 4.3. Immunosensors

Immunosensing is a method of biosensing where the sensitive detection is based on the specific binding between an antigen and an antibody to form a stable complex. Immunosensors combine the advantages of good sensitivity and high selectivity. Furthermore, they allow the progress of immunoreactions on detector surfaces to be followed in real-time [40]. A crucial step in the design of immunosensors is the immobilization of antibodies on the surface of the electrode. Since CPs can be easily oxidized and reduced, their zeta potential and surface charge density depend on pH and ionic strength, allowing for tailored electrostatic interactions between charged proteins affecting their adsorption [41].

A three-dimensional macroporous PAn-based electrode was developed for the sensitive detection of alpha-fetoprotein, which can be associated with several conditions, including hepatocellular carcinoma, metastatic disease affecting the liver, nonseminomatous germ cell tumor, or yolk sac tumor. A PAn-based substrate provided an excellent platform for the immobilization of alpha-fetoprotein antibody and the resulting biosensor achieved good linearity in the wide range from 0.01 to 1000 pg mL^−1^ [42]. In another study, conducting polythiols and gold nanoparticles (AuNPs) were used to develop a biosensor sensitive to the carcinoembryonic antigen (CEA), a biomarker for colorectal cancer [43]. The results revealed a good amperometric response to CEA, with a linear range from 1 fg mL^−1^ to 10 ng mL^−1^, and an LOD of 0.015 fg mL^−1^. Furthermore, the high applicability of AuNPs in electrochemical cancer detection was confirmed, as supported by further studies [44,45].

**Scheme 7:**
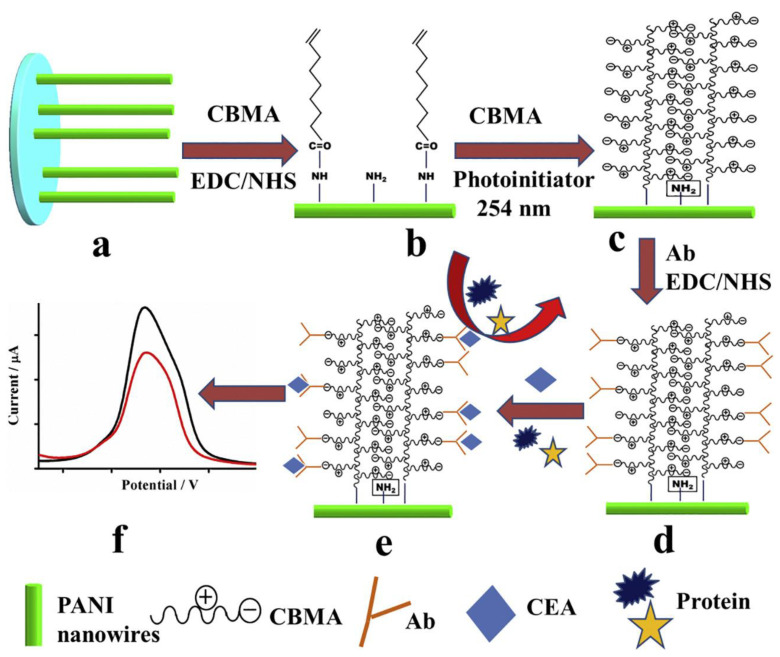
Schematic illustration of the construction process of the CEA biosensor: (**a**) Polyaniline (PANI) nanowires electrodeposited onto the GCE, (**b**) covalently bound CBMA on PANI nanowires, (**c**) a photopolymerized polyCBMA layer on PANI nanowire, (**d**) the immobilization of CEA antibody on the polyCBMA/PANI composite, (**e**) specific CEA capturing and the antifouling towards nonspecific protein, (**f**) DPV current signal recording. Reprinted with permission from [46]. Copyright (2018) Elsevier.

The electrochemical detection of CEA was also studied by Wang and Hui [46], who successfully designed a zwitterionic poly(carboxybetaine methacrylate) (polyCBMA) composite with conducting PAn nanowires (Scheme 7). PolyCBMA exhibited good antifouling properties, which in combination with PAn provided an excellent immunosensor, characterized by low fouling properties, that prevented non-specific protein adsorption. The biosensor was used to quantify the presence of CEA in an undiluted human blood serum sample, demonstrating the practical usability of the system in clinical analysis. Another antifouling electrochemical biosensing platform was fabricated from PEDOT and a multifunctional peptide with a sequence of CAEAEPPPPQEQKQEQK and used for the selective and sensitive quantification of CA 15-3 in breast cancer patients [47]. During the polymerization process, the peptide was doped within PEDOT to achieve a negative charge on the surface of the sensor. The antifouling property of the modified sensor decreased by only ~12% after using the same sensor for up to 30 days. These long-term, stable antifouling properties are ideal for implantable sensor devices.

### 4.4. Detection of Enzymes

**Scheme 8:**
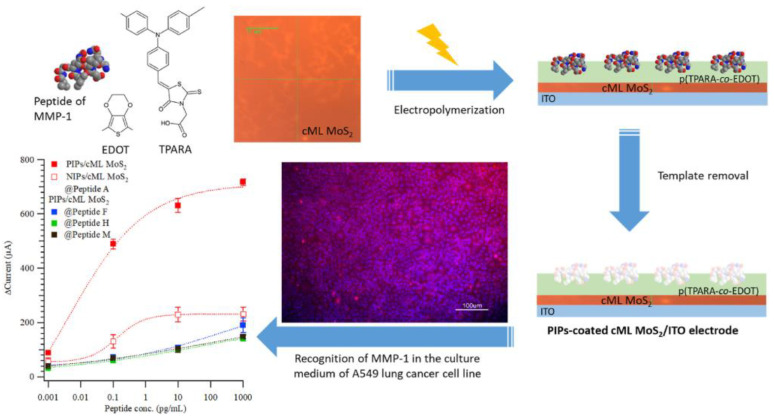
Preparation and recognition of MMP-1 peptide-imprinted poly(TPARA-*co*-EDOT)-coated cML MoS_2_/ITO electrodes in the culture medium of A549 lung cancer cell line. Reprinted with permission from [48]. Copyright (2022) Elsevier.

Since cancer dysregulates metabolic activity and reprograms affected cells, the presence of increased levels of certain enzymes in body fluids may be a sign of disease. Poly(triphenylamine rhodanine-3-acetic acid-co-3,4-ethoxylene dioxy thiophene)s incorporated with molybdenum disulfide were reported for the monitoring of matrix metalloproteinase-1 (MMP-1), which is associated with lung cancer [48]. Electrode modification was carried out by incorporating a CP on the surface of molybdenum disulfide by a peptide-imprinted electropolymerization process using MMP-1 peptides as templates (Scheme 8). As-formed thin film electrodes provided the monitoring of MMP-1 at 1 pg mL^−1^ with a linear range of 1–10 pg mL^−1^. The validation of the performance of a fabricated biosensor was performed for a human lung cancer cell line (A549), in which an MMP-1 concentration of 800 ng mL^−1^ was found. This method showed 95% accuracy when compared with the conventional enzyme-linked immunosorbent assay (ELISA) method. A PPy-based biosensor was reported for the electrochemical detection of another enzyme, human autocrine motility factor-phosphosglucose isomerase, which is considered to be a metastatic biomarker in human plasma. Amperometric quantification was performed in the linear range of 1 pM-1μM with an LOD of 43 fM in a phosphate buffer solution [49].

### 4.5. Detection of Neurotransmitters

In the past decades, many research studies have reported on the regulatory role of neurotransmitters in physiological and pathological functions of tissues and organs. Notably, emerging data suggest that cancer cells use neurotransmitter-initiated signaling pathways to activate uncontrolled proliferation and dissemination. In addition, neurotransmitters can affect immune cells and endothelial cells in the tumor microenvironment to promote tumor progression [50]. Chung et al. [51] developed a CP-based palladium complex for the electrochemical detection of the neurotransmitters dopamine and serotonin in the cancer cell lines. The proposed sensor was fabricated using a poly2,2:5,2-terthiophene-3-(p-benzoic acid) layer anchored with the Pd(C_2_H_4_N_2_S_2_)_2_ complex on the reduced graphene oxide substrate decorated with AuNPs. To achieve maximum sensitivity of the sensor, various parameters such as the concentration of AuNPs-reduced graphene oxide, the number of electropolymerization cycles, and the immobilization of Pd complex were optimized. The LOD of serotonin and dopamine was achieved as 2.5 nM and 24 nM, respectively, and the reliability of the sensor was evaluated in both normal and breast cancer cells. The results showed that the serotonin level released in breast cancer cells was 3.1 times higher than in normal cells. Although more studies on the application of neurotransmitters as cancer biomarkers are inevitable, CPs and their metallic and non-metallic composites appear to be excellent candidates for the design of such biosensors, since they have been successfully used for the monitoring of neurotransmitters such as glutamate, aspartate, tyrosine, epinephrine, norepinephrine, dopamine, serotonin, histamine, choline, and acetylcholine [52].

### 4.6. Cell Capture

An interesting alternative to the detection of molecular and macromolecular biomarkers is the possibility to capture and analyze whole cells, particularly circulating tumour cells (CTC) released from cancerous cells in the primary and metastatic stages. CP nanostructures-based cell capture systems were first reported by Sekine et al. [53], who used PEDOT-COOH nanodots fabricated through an electropolymerization process on the surface of indium tin oxide (ITO)-coated glass electrodes. Due to the synergistic effects of ligand–receptor interaction, nanostructure adaptation, and mechanical property adjustment, modified surfaces were able to capture CTC through epithelial cell adhesion molecule (EpCAM) antibodies bioconjugated to PEDOT-COOH.

**Scheme 9:**
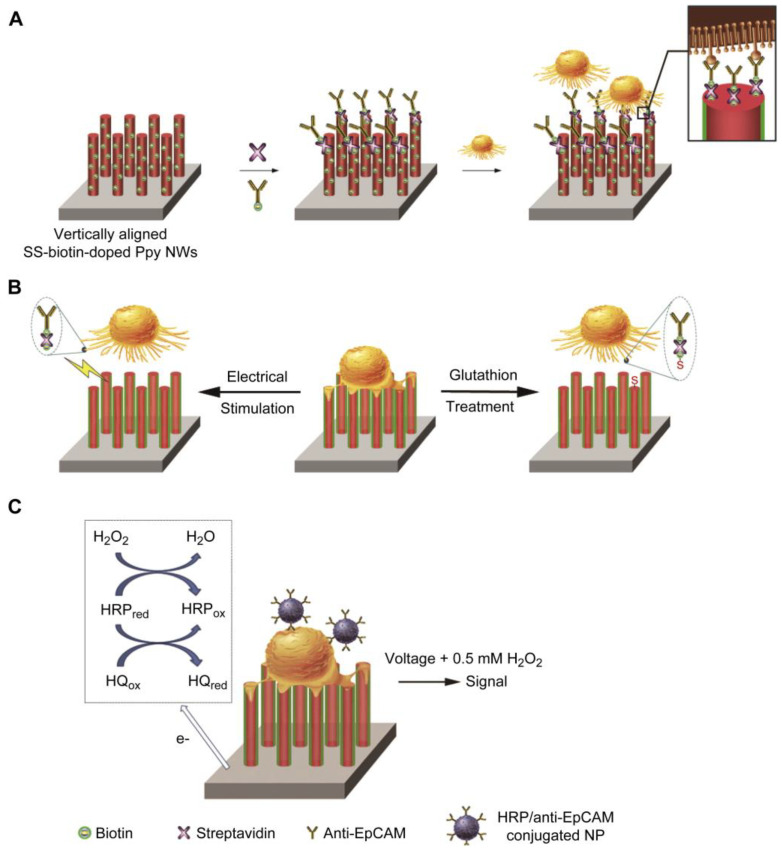
(**A**) Fabrication of anti-EpCAM-immobilized SS-biotin-doped PPy nanowires (anti-EpCAM-SS-biotin-Ppy NWs) of 200-nm diameter for capture, release, and in situ quantification of cancer cells. (**B**) The dual electrical simulation (ES)- and glutathione (GSH)-responsive system for the release of the captured cells. (**C**) On-site electrochemical detection of the captured cancer cells by a nanoparticle-based signal amplification strategy. Reprinted with permission from [54]. Copyright (2014) Elsevier.

In another study, PPy nanowires doped with biotin were used to capture, release and quantify CTCs (Scheme 9) [54]. Electrochemical detection of the captured cancer cells using a PPy nanowire platform with horseradish peroxidase-labeled and conjugated nanoparticles showed high sensitivity and specificity. Luo and co-workers [55] developed the electrodeposited PEDOT and a multifunctional peptide-based composite to avoid the biofouling effect in the detection of CTCs. The designed peptide showed antifouling properties in complex biological media and provided special recognition for breast cancer cells (MCF-7). The electrodeposited PEDOT formed an electroactive surface suitable for the detection of cancer cells in blood without dilution and purification. CP-based microfluidic devices and composites are already available in the literature for the capturing and detection of CTCs [56,57,58,59]. Recently, a conducting ter-polymer-coated carbon cloth designed by Ashraf et al. [60] was used for the capture and release of extracellular vesicles originating from both MCF-7 and SKBR-3 breast cancer cell lines. Cell capturing was carried out with the aid of a substrate conjugated antibody, and the performance of the developed system was validated in real samples.

### 4.7. Multiplex Biomarkers Detection

Due to the complexity of the biological systems, an accurate diagnosis is not usually possible by the analysis of a single biomarker alone. Consequently, an improved accuracy for cancer detection is achievable through the simultaneous detection of multiple biomarkers. There are few reports on the fabrication of biosensors for multiple biomarkers based on conducting polymer matrices. For instance, a PPy-modified gold electrode was reported for the electrochemical detection of the oral cancer biomarkers interleukin (IL)-8 mRNA and IL-8 protein. Under optimized conditions, the LOD of salivary IL-8 mRNA and IL-8 protein reached 3.9 fM and 7.4 pg mL^−1^, respectively. The accuracy of the sensor was found to be 90%, and similar results were obtained when compared with conventional clinical methods such as ELISA [61]. On the other hand, the simultaneous electrochemical detection of alpha-fetoprotein (AFP) and CEA was achieved by fabricating redox-active species, poly(o-phenylenediamine) (POPD)/Au nanocomposite and poly(vinyl ferrocene-2-aminothiophenol) (poly(VFc-ATP))/Au nanocomposite, where HAuCl_4_ was chosen as the oxidant [62]. AuNPs-decorated polymers exhibited the successful immobilization of anti-CEA and anti-AFP as the immunosensing probes, which enabled the sensitive detection of AFP and CEA with an LOD of 0.003 ng mL^−1^ and 0.006 ng mL^−1^, respectively.

## 5. CP-Based Electrochemical Sensors for Cancer Diagnosis: Comparison

In the previous section, a detailed discussion of the available electrochemical biosensors based on CPs was presented. An overview of the reported results, including electrode material, biomarkers, sensing technique, range of linearity, limit of detection, and biomarker source is presented in Table 1.

## 6. Summary and Outlooks

The development of biosensors for the early detection of cancer biomarkers has been reported in recent years by focusing on the sensitive detection of DNAs, miRNAs, enzymes, proteins, etc., related to the presence of cancerous cells in the human body. Although electrochemical biosensors are able to provide rapid and continuous detection, the practicability of the electrochemical methods is restricted due to biofouling effects and thereby limited stability. Researchers are currently devoting great efforts to achieve the maximum sensitivity and selectivity of biosensors and to avoid the biofouling effect from proteins and biomolecules present in blood serum samples during practical applications.

CPs and their composites have been the subject of intensive studies, particularly in the development of electrochemical sensors, for the ultrasensitive detection of cancer biomarkers. Their large surface area, porosity, and the presence of functional groups provide CPs with binding sites suitable for capturing biomarkers, as well as enabling sensitive and easy detection. Consequently, CPs have been found to act as excellent electrode materials able to immobilize a wide range of molecular recognition elements allowing for the ultrasensitive recognition (with LOD as low as 0.5 fM) of DNAs, miRNAs, proteins, enzymes, neurotransmitters, as well as whole cancer cells (LOD of 10 cells). More importantly, functionalized CP surfaces have been found to limit the biofouling effect, allowing for sensitive detection in real patient samples. The anti-fouling property of a sensor material is a supreme character in the practical applications of biomarker detection. Similarly, from the development of biosensors for a single biomarker, researchers have now reached the stage where a single biosensor can detect multiple biomarkers at a time. In this way, it is possible to provide the detailed encryption of the cancerous initiation and growth. Additionally, CPs furnish the direct cell capturing and sensing, which will make the cancer diagnosis more accurate and efficient. Nevertheless, apart from the laboratory methods, no devices are reported elsewhere for the point of care diagnosis of cancer biomarkers. The fabrication of stable, sensitive, selective and antifouling cancer biomarker biosensing devices, particularly those based on CPs, is expected to be a positive new development in the field of cancer diagnosis to replace the conventional analytical methods. 

## Figures and Tables

**Table 1:** Comparative study on the available CP-based electrochemical biosensors for the detection of various cancer biomarkers (alpha-fetoprotein: AFP, carcinoembryonic antigen: CEA, circulating tumour cells: CTC; interleukin: IL). Abbreviations: chronoamperometry, CA; cyclic voltammetry: CV; differential pulse voltammetry: DPV; electrochemical impedance spectroscopy: EIS; glassy carbon electrode: GCE; gold electrode: AuE; gold nanoparticles: AuNPs; graphene oxide: GO; indium tin oxide: ITO; multi-walled carbon nanotubes: MWCNTs; polyaniline: PAn; poly(3,4-ethylenedioxythiophene): PEDOT; polypyrrole: PPy; screen printed electrode: SPE; square wave voltammetry: SWV.

Electrode Material	Biomarker	Sensing Technique	Linear Range	Limit of Detection	Real Sample	Ref.
GCE/PEDOT/polypeptide	BRCA1	DPV	1 × 10^−5^–1 nM	3.4 × 10^−6^ nM	Serum	[33]
GCE/GO/PPY	BRCA1	DPV	1 × 10^−7^–100 nM	3 × 10^−6^ nM	Serum	[34]
AuE/PAn	miRNA	EIS	1 × 10^−6^–5 × 10^−3^ nM	5 × 10^−7^ nM	Cancer cells & blood	[36]
RuO_2_-templated electropolymerized PAn	miRNA	SWV	5 × 10^−7^–2 × 10^−3^ nM	2 × 10^−6^ nM	Lung cancer cells	[37]
GCE/o-MWCNTs/Poly(5-hydroxy-1,4-naphthoquinone)	miR-141	SWV	1 × 10^−6^–10 nM	8 × 10^−6^ nM	Serum	[38]
PAn nanowires deposited after hybridization	miRNA	Conductance measurements	1 × 10^−7^–2 × 10^−2^ nM	5 × 10^−6^ nM	RNA extracted from Hela cells and lung cancer cells	[39]
GCE/PAn	AFP	DPV	0.01–1 ng mL^−1^	3.7 × 10^−6^ ng mL^−1^	Serum	[42]
Polythiols/AuNPs	CEA	DPV	1 × 10^−6^–10 ng mL^−1^	15 × 10^−9^ ng mL^−1^	Serum	[43]
GCE/Poly(carboxybetaine methacrylate)/PAn	CEA	DPV	1 × 10^−5^–0.1 ng mL^−1^	3.05 × 10^−6^ ng mL^−1^	Serum	[46]
GCE/AuNPs/PEDOT/Peptide	CA 15-3	DPV	1 × 10^6^–1 × 10^12^ nU mL^−1^	3.2 × 10^2^ nU mL^−1^	Serum	[47]
ITO/Poly(triphenylamine rhodanine-3-acetic acid-co-3,4-ethoxylene dioxy thiophene)/MoS_2_/Peptide	Matrix metalloproteinase-1	CV	1 × 10^−3^–1 × 10^−2^ ng mL^−1^	1 × 10^−3^ ng mL^−1^	Lung cancer cells	[48]
AuE/PPy/Polydendrimer	Phosphosglucose isomerase	CA	1 × 10^−3^–1 × 10^3^ nM	4.3 × 10^−5^ nM	Human plasma	[49]
SPE/Au/reduced GO/ Poly(2,2:5,2- terthiophene-3-(p-benzoic acid))/Pd	Serotonine & dopamine	DPV	20–2 × 10^5^ nM & 100–2 × 10^5^ nM	2.5 & 24 nM	Breast cancer cells	[51]
Biotin-doped PPy nanowires	CTC	CA	10–10^4^ cells	10 cells	Cancer cells	[54]
PEDOT/peptide	CTC	DPV	50–10^6^ cells mL^−1^	17 cells mL^–1^	Breast cancer cells	[55]
AuE/PPy	IL-8 mRNA & IL-8 protein	CA	5 × 10^−6^–5 × 10^−4^ nM & 1 × 10^−4^–12.5 ng mL^−1^	3.9 × 10^−3^ nM & 7.4 × 10^−3^ ng mL^−1^	Saliva	[61]
Poly(vinyl ferrocene -2-aminothiophenol)/Au	AFP & CEA	DPV	0.01–100 ng mL^−1^	3 × 10^−3^ & 6 × 10^−3^ ng mL^−1^	Serum	[62]

## Data Availability

Not applicable.
